# miR-106a-5p Inhibits the Proliferation and Migration of Astrocytoma Cells and Promotes Apoptosis by Targeting FASTK

**DOI:** 10.1371/journal.pone.0072390

**Published:** 2013-08-27

**Authors:** Feng Zhi, Guangxin Zhou, Naiyuan Shao, Xiwei Xia, Yimin Shi, Qiang Wang, Yi Zhang, Rong Wang, Lian Xue, Suinuan Wang, Sujia Wu, Ya Peng, Yilin Yang

**Affiliations:** 1 Modern Medical Research Center, Third Affiliated Hospital of Soochow University, Changzhou, Jiangsu, China; 2 Department of Neurosurgery, Third Affiliated Hospital of Soochow University, Changzhou, Jiangsu, China; 3 Department of Orthopedics, Jinling Hospital, School of Medicine, Nanjing University, Nanjing, Jiangsu, China; The Ohio State University, United States of America

## Abstract

Astrocytomas are common malignant intracranial tumors that comprise the majority of adult primary central nervous system tumors. MicroRNAs (miRNAs) are small, non-coding RNAs (20–24 nucleotides) that post-transcriptionally modulate gene expression by negatively regulating the stability or translational efficiency of their target mRNAs. In our previous studies, we found that the downregulation of miR-106a-5p in astrocytomas is associated with poor prognosis. However, its specific gene target(s) and underlying functional mechanism(s) in astrocytomas remain unclear. In this study, we used mRNA microarray experiments to measure global mRNA expression in the presence of increased or decreased miR-106a-5p levels. We then performed bioinformatics analysis based on multiple target prediction algorithms to obtain candidate target genes that were further validated by computational predictions, western blot analysis, quantitative real-time PCR, and the luciferase reporter assay. Fas-activated serine/threonine kinase (FASTK) was identified as a direct target of miR-106a-5p. In human astrocytomas, miR-106a-5p is downregulated and negatively associated with clinical staging, whereas FASTK is upregulated and positively associated with advanced clinical stages, at both the protein and mRNA levels. Furthermore, Kaplan-Meier analysis revealed that the reduced expression of miR-106a-5p or the increased expression of FASTK is significantly associated with poor survival outcome. These results further supported the finding that FASTK is a direct target gene of miR-106a-5p. Next, we explored the function of miR-106a-5p and FASTK during astrocytoma progression. Through gain-of-function and loss-of-function studies, we demonstrated that miR-106a-5p can significantly inhibit cell proliferation and migration and can promote cell apoptosis *in vitro*. The knockdown of FASTK induced similar effects on astrocytoma cells as those induced by the overexpression of miR-106a-5p. These observations suggest that miR-106a-5p functions as a tumor suppressor during the development of astrocytomas by targeting FASTK.

## Introduction

Astrocytomas are the most common primary brain tumors in the central nervous system [1]. Despite new biological insights and therapeutic advances, the general prognosis for astrocytoma patients remains poor, particularly in patients with high-grade astrocytomas, for which the median survival time is only 15 months [2]. The pathogenesis of astrocytomas is complex and involves the aberrant activation of oncogenes and the inactivation of tumor suppressor genes, such as *EGFR* and *PTEN* [3], [4]. Increasing numbers of genetic and molecular aberrations, which are the causes and consequences of deregulated intracellular signaling networks, have been correlated with the development of astrocytomas. However, these alterations have not been fully elucidated.

MicroRNAs (miRNAs) are endogenous non-coding RNAs consisting of 19 to 24 nucleotides, which modulate gene expression by base pairing with complementary sites on target mRNAs, blocking translation or triggering the degradation of the target mRNAs [5]. Increasing evidence has revealed that miRNAs are aberrantly expressed in a wide variety of human cancers and exhibit a causal role in tumorigenesis [6], [7]. In our previous studies, we found that miR-106a-5p is significantly downregulated in astrocytomas. Furthermore, the reduced expression of miR-106a-5p is associated with poor survival outcome, suggesting that miR-106a-5p elicits a tumor suppressive role during astrocytoma development and/or progression [8]. miR-106a-5p belongs to the miR-17 family, which includes miR-17-5p, miR-20a, miR-20b, miR-106a-5p, miR-106b and miR-93. The miR-17 family is divided into three clusters according to consensus seed regions, and miR-106a-5p is a member of the miR-106a-363 cluster, which is located on Xq26.2. miR-106a-5p is highly expressed in gastric [9], [10], [11], [12], [13], breast [14], [15], colorectal [16] and non-small cell lung cancers [17]. It is also expressed at lower levels in squamous cell carcinomas [18], colon cancers [19] and gliomas [8], [20]. Whether miR-106a-5p is a tumor suppressive or oncogenic miRNA remains controversial, and the regulatory mechanism underlying miR-106a-5p-mediated function remains to be elucidated in different cancers. Recently, miR-106a-5p was found through bioinformatics prediction followed by experimental validation to inhibit glioma cell growth by targeting the transcription factor E2F1 [20]. However, the downstream effectors of miR-106a-5p in astrocytomas remain elusive, and thus, further research is required to fully understand its contributions to this malignancy.

In order to identify the total transcripts regulated by miR-106a-5p directly or indirectly, we first measured the global mRNA expression change through mRNA microarray by overexpressing or knocking down miR-106a-5p in cancer cells. Only those genes that were inversely expressed relative to miR-106a-5p expression change were chosen. Next, we combined bioinformatics programs to select candidate miR-106a-5p targets from the differentially regulated genes to refine the number of miR-106a-5p targets. Then we validated the miR-106a-5p target gene through western blot analysis, quantitative real-time PCR and luciferase reporter assay. Fas-activated serine/threonine kinase (FASTK) was identified as a direct target of miR-106a-5p. Subsequently, we asked whether miR-106a-5p or FASTK expression levels represented specific molecular signatures for subsets of astrocytomas. In total, 84 patients with astrocytomas and normal adjacent tissues (NATs) from 20 astrocytoma patients were enrolled in this study. We found that miR-106a-5p expression is significantly downregulated in astrocytoma tissues and is negatively associated with advanced clinical staging, whereas FASTK exhibited the opposite expression pattern. Using Kaplan-Meier survival analysis, we showed that the low expression of miR-106a-5p or the increased expression of FASTK is significantly associated with poor survival outcome in astrocytoma patients. Finally, we investigated the potential function of miR-106a-5p and FASTK in the development and progression of astrocytomas. We found that miR-106a-5p significantly suppressed cell proliferation and migration and promoted cell apoptosis *in vitro*. The knockdown of FASTK induced similar effects on astrocytoma cells as those induced by miR-106a-5p. These results elucidate the underlying mechanism by which miR-106a-5p inhibits astrocytoma development and progression.

## Materials and Methods

### Human Tissue Samples

Surgically excised tumor specimens from 84 patients with astrocytomas and normal adjacent tissues (NATs) from 20 astrocytoma patients were collected in the Department of Neurosurgery of the Third Affiliated Hospital of Soochow University, China. The cases included 5 patients with grade I, 26 with grade II, 33 with grade III and 20 patients with grade IV astrocytomas. Histological grading was performed on the basis of the World Health Organization (WHO) criteria. Collected tissues were immediately snap-frozen in liquid nitrogen and stored at −80°C. The demographic and clinical features of the patients are listed in Table S1 in File S1. Written informed consent was obtained from all patients or their representatives before the study, which was approved by the Research Ethics Board of the Third Affiliated Hospital of Soochow University.

### Cell Culture

The human astrocytoma cell line U251 was purchased from Cell Resource Centre of the Shanghai Institutes for Biological Sciences of the Chinese Academy of Sciences. All of the cells were cultured in Dulbecco’s Modified Eagle’s Medium (Invitrogen, USA) supplemented with 10% fetal bovine serum (Hyclone, USA) in a humidified 37°C incubator that was maintained at 5% CO_2_.

### Overexpression and Knockdown of miR-106a-5p

The oligonucleotide miR-106a-5p mimic (pre-miR-106a-5p), mimic negative control (pre-ncRNA), miR-106a-5p inhibitor (anti-miR-106a-5p) and the inhibitor negative control (anti-ncRNA) were purchased from GenePharma (Shanghai, China). U251 cells were seeded onto 6-well plates and were transfected the following day using Lipofectamine 2000 (Invitrogen, Carlsbad, CA, USA) according to the manufacturer’s protocol. miR-106a-5p overexpression was achieved by transfecting cells with pre-miR-106a-5p, whereas miR-106a-5p knockdown was achieved by transfecting cells with anti-miR-106a-5p. The pre-ncRNA and anti-ncRNA served as negative controls. For each well, equal concentrations (100 pmol) of pre-ncRNA, pre-miR-106a-5p, anti-ncRNA or anti-miR-106a-5p were added. The cells were harvested at 24 h after transfection.

### RNA Isolation and Quantitative Real-time PCR

Total RNA was extracted from cultured cells or clinical samples using TRIzol Reagent (Invitrogen) according to the manufacturer’s protocols. The RNA molecules were then treated with RNase-free DNase (TaKaRa, Dalian, China) using a standardized protocol. The levels of mature miR-106a-5p were quantified using Taqman microRNA probes (Applied Biosystems) as previously reported [21]. Briefly, 2 µg of total RNA was reverse-transcribed to cDNA using an AMV reverse transcriptase (TaKaRa, Dalian, China) and a stem-loop primer (Applied Biosystems). The mixture was incubated at 16°C for 15 min, 42°C for 60 min and 85°C for 5 min to generate a library of miRNA cDNAs. Quantitative real-time PCR (qRT-PCR) was performed using a TaqMan PCR kit on an Applied Biosystems 7500 Sequence Detection System (Applied Biosystems, Foster City, CA, USA). All of the reactions were performed in triplicate. For normalization, the U6 small nuclear RNA was used as a control. The relative amount of miR-106a-5p to internal control U6 transcript was calculated using the equation 2^−△CT^ in which △C_T_ = C_T__miR-106a-5p_ − C_T__U6_. For the analysis of FASTK and β-actin, qRT-PCR was performed on an Applied Biosystems 7500 Sequence Detection System (Applied Biosystems, Foster City, CA, USA) using SYBR green dye (Invitrogen). First, 1 µg of total RNA was reverse transcribed to cDNA with oligodT (TaKaRa, Dalian, China). Then, the reactions were incubated in a 96-well plate at 95°C for 5 min, followed by 40 cycles of 95°C for 30 s, 60°C for 30 s, and 72°C for 30 s. All of the reactions were performed in triplicate. The sequences of the sense and antisense primers used for the amplification of FASTK and β-actin were as follows: FASTK (sense): 5′- GGTGGGCAAGGGTTGGAAG-3′, FASTK (antisense): 5′- CTGCTGAGTTGCGTTTCCT-3′, β-actin (sense): 5′-AGGGAAATCGTGCGTGAC-3′, and β-actin (antisense): 5′-CGCTCATTGCCGATAGTG-3′.

### mRNA Microarray Procedure

Briefly, U251 cells were transfected with pre-ncRNA, pre-miR-106a-5p, anti-ncRNA or anti-miR-106a-5p using Lipofectamine 2000. The cells were harvested at 24 h after transfection, and total RNA was extracted from the cultured cells using TRIzol Reagent. The mRNA microarray was performed at CapitalBio Corporation (Beijing, China). The microarray (Probe length 60–80-mer, 12×135 K Format) containing 44,987 probe sets was provided by Roche-NimbleGen. For each sample, 1 µg of total RNA was treated with the CapitalBio cRNA Amplification and Labeling Kit (CapitalBio, China) according to the manufacturer’s protocols. After first strand reverse transcription with a T7 oligo(dT) primer and second strand synthesis followed by purification, the synthesized cDNAs were transcribed to cRNAs *in vitro* using the T7 Enzyme Mix. Next, 5 µg of purified cRNAs was reverse transcribed with random primers. Labeled cDNA molecules were generated by subsequent Klenow Fragment Polymerase labeling with Cy3-dCTP (GE Healthcare, Cat. No. PA 53021, USA). After purification and drying, the labeled cDNAs were then dissolved in 80 µl hybridization buffer (3×SSC, 0.2% SDS, 5×Denhart’s, 25% formamide) and hybridized with the arrays overnight at 42°C on a hybridization system 12 (Roche NimbleGen, USA). The arrays were then washed and dried. The fluorescence intensity was measured using a NimbleGen MS 200 Microarray Scanner. The data were extracted from scanned images using NimbleScan v2.6 software. Quantile normalization RMA (Robust Multi-Array) analysis was performed to generate gene expression values. The differences between the 2 groups were analyzed using the SAM (Significance Analysis of Microarrays) method with SAMR software version 3.02 [22]. Differentially expressed genes (DEGs) were selected with a False Discovery Rate (FDR) <5%. The microarray data has been deposited in NCBI Gene Expression Omnibus (GEO) database under accession number GSE47737.

### miR-106a-5p Target Prediction

The analysis of microRNA predicted targets was determined using the algorithms from TargetScan [23], PicTar [24] and microRNA.org [25].

### Western Blotting

Total cellular protein was isolated using RIPA lysis buffer (Sigma-Aldrich Inc., St Louis, MO) at 48 h after transfection. Western blotting analysis was performed using conventional protocols as previously described [26]. Briefly, the rabbit monoclonal β-actin antibody (Cell Signaling Technology, USA), rabbit polyclonal FASTK antibody (Abcam, UK), and the secondary rabbit IgG-HRP (Sigma, USA) were applied at 1∶1,000, 1∶500, and 1∶5,000 dilutions, respectively. The Quantity One analysis program (Bio-Rad, USA) was used to obtain the quantitative data.

### Luciferase Assay

The entire human FASTK 3′-untranslated region (3′-UTR) was amplified by PCR using human genomic DNA as a template. The PCR products were inserted into the p-MIR-report plasmid (Ambion). Efficient insertion was confirmed by sequencing. For the luciferase reporter assays, cells were cultured in 6-well plates, and each well was transfected with 2 µg of firefly luciferase reporter plasmid, 2 µg of β-galactosidase expression vector (Ambion), and equal amounts of pre-ncRNA, pre-miR-106a-5p, anti-ncRNA, or anti-miR-106a-5p using Lipofectamine 2000 (Invitrogen). The β-galactosidase vector was used as a transfection control. At 24 h post-transfection, cells were assayed using luciferase assay kits (Promega, Madison, WI, USA). The data depicted represent three independent experiments performed on different days.

### siRNA Interference Assay

Three siRNA sequences targeting different sites of the human FASTK cDNA (si-FASTK) were designed and synthesized by Invitrogen (Invitrogen, USA). A scrambled siRNA (si-NC) that could not target the human FASTK cDNA was included as a negative control. The siRNAs were as follows: siRNA-1 (HSS145880), siRNA-2 (HSS173974), siRNA-3 (HSS145881). The siRNA was transfected into U251 cells using Lipofectamine 2000 (Invitrogen) according to the manufacturer’s protocol. Total RNA was isolated at 24 h post-transfection. The expression levels of FASTK mRNA were assessed by qRT-PCR. Total cellular protein was isolated at 48 h after transfection. The expression levels of FASTK protein were assessed by western blot. The sequence with the best interfering effect was selected and used in further studies.

### Cell Viability Assay

U251 cells in logarithmic growth were transfected with pre-miR-106a-5p or si-FASTK with their corresponding controls. 24 hours after transfection, the cells (approximately 5×10^3^) were seeded into 96-well culture plates for 12, 24, 48, 72, and 96 h. Next, 20 µl MTT (5 mg/mL) was added to each test well and incubated for 4 h at 37°C. The supernatant was discarded subsequently, and 150 µl of dimethyl sulfoxide (DMSO) was added to each well to solubilize the crystals for 10 min at room temperature. The optical density (OD) was measured at a wavelength of 570 nm.

### Cell Migration Assay

The ability of U251 cells to migrate was assayed by the transwell crystal violate method using Transwell inserts (24-well insert, pore size 8 µm; Corning, Inc., Corning, NY). Cells transfected with pre-miR-106a-5p, si-FASTK or their corresponding negative controls were suspended in serum-free DMEM culture medium at a concentration of 4×10^5^ cells/mL and then added to the upper chamber (4×10^4^ cells/well). Simultaneously, the lower chambers were filled with 10% FBS as a chemoattractant, and the cells were incubated in the chambers for 48 h. At the end of the experiments, the cells on the upper surface of the membranes were removed using a cotton swab, and the cells on the lower surface were fixed and stained with 0.1% crystal violet. Five visual fields of each insert were randomly chosen and counted under a microscope (IX71, Olympus, Japan). The software used to count migrated and non-migrated cells was Image-Pro Insight (Version 8.0.21). The mean number of migrating or invading cells was expressed as a percentage relative to the control.

### Cell Apoptosis Assay

Cells transfected with pre-miR-106a-5p, si-FASTK or with their corresponding negative controls were harvested at 24 h after transfection and washed in cold PBS. Combined Annexin V and propidium iodide staining was performed using the Annexin V-FITC apoptosis detection kit (BD Biosciences, San Jose, CA), according to the manufacturer’s protocol. After flow cytometric analysis of the cells, apoptosis profiles were obtained with the Cell Quest Pro software.

### Statistical Analysis

Data shown are presented as the mean ± SD of at least three independent experiments. The differences were considered statistically significant when p<0.05. The survival curve was estimated using the Kaplan-Meier method in SPSS 13.0, and the resulting curves were compared using the log-rank test.

## Results

### Identification of Transcripts Regulated by miR-106a-5p using Microarray Analysis

**Figure 1:**
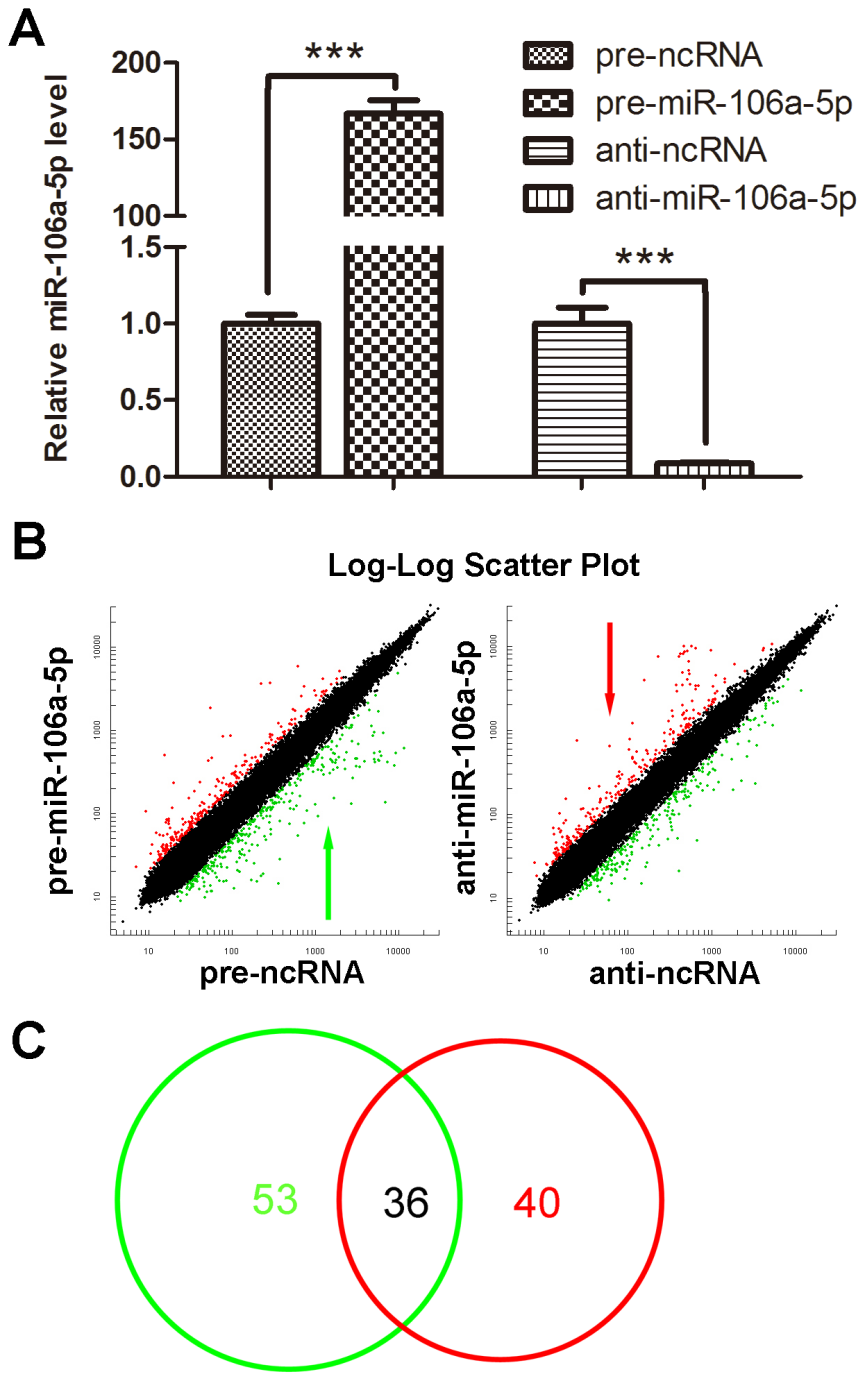
Differentially regulated genes in cells with increased or decreased expression of miR-106a-5p. (A) Overexpression or knockdown of miR-106a-5p. U251 cells were seeded into 6-well plates and transfected the following day using Lipofectamine 2000. For each well, 100 pmol of pre-ncRNA, pre-miR-106a-5p, anti-ncRNA or anti-miR-106a-5p was transfected. The intercellular levels of miR-106a-5p were evaluated by qRT-PCR at 24 h after transfection. For comparison, the expression levels of miR-106a-5p in pre-ncRNA- or anti-ncRNA-transfected cells were arbitrarily set at 1. The results are presented as the mean ± SD of three independent experiments (*** p<0.001). (B) The scatter plot of altered genes that were inversely expressed with increased or decreased expression of miR-106a-5p. Left: downregulated genes when miR-106a-5p is upregulated; Right: upregulated genes when miR-106a-5p is downregulated. (C) A Venn diagram of the overlap of altered genes with increased or decreased miR-106a-5p expression. The differentially expressed genes are depicted as two overlapping circles. The green circle indicates the number of genes that are downregulated when miR-106a-5p is upregulated, whereas the red circle indicates the number of genes that are upregulated when miR-106a-5p is downregulated. The number in the overlapping area indicates the number of mRNAs that belong to the intersecting sets.

The transcripts regulated both directly and indirectly by miR-106a-5p were identified using a previously reported method [27]. We first transfected U251 cells with equal concentrations of pre-ncRNA, pre-miR-106a-5p, anti-ncRNA or anti-miR-106a-5p. We then surveyed potential genes that were inversely expressed relative to miR-106a-5p using mRNA microarray analysis. As shown in Figure 1A, the expression of miR-106a-5p was significantly increased by the introduction of pre-miR-106a-5p, whereas anti-miR-106a-5p abolished the miR-106a-5p levels in U251 cells. The mRNA microarray profiles clearly showed differential mRNA expression patterns among pre-miR-106a-5p- and anti-miR-106a-5p-transfected cells compared with their corresponding control transfectants (Figure 1B). In total, 89 genes were downregulated (mean fold-change ≤0.5) in miR-106a-5p-overexpressing cells, whereas 76 genes were upregulated (mean fold-change ≥2.0) in miR-106a-5p-downregulated cells (Figure 1C). To reduce the false positives and to obtain a more accurate assessment of the genuine miR-106a-5p targets, only the mRNAs that were present in both the pre-miR-106a-5p- and the anti-miR-106a-5p-transfected groups were considered as candidate miR-106a-5p targets. A set of 36 genes was identified as candidate miR-106a-5p targets (Figure 1C and Table 1) In order to validate the specificity of pre-miR-106a-5p and anti-miR-106a-5p and their off target effects, we transfected U251 cells with equal amounts of pre-ncRNA, pre-miR-106a-5p, anti-ncRNA or anti-miR-106a-5p, and the expression level of miR-106b, another member of the miR-106 family whose sequence is most similar to miR-106a-5p, was assessed by quantitative RT-PCR assay. As can be seen in Figure S1 in File S1, while miR-106a-5p was significantly upregulated by transfection of pre-miR-106a-5p and downregulated by transfection of anti-miR-106a-5p, the expression levels of miR-106b were unaffected. These results demonstrate that the endogenous level of miR-106a-5p can be specifically manipulated by miR-106a-5p mimics or inhibitors, ant this approach have no obvious off target effects.

**Table 1:** mRNAs present in both the pre-miR-106a-5p- and the anti-miR-106a-5p-transfected groups.

Gene Name	Fold-Change (Up)	Fold-Change (Down)	Gene Name	Fold-Change (Up)	Fold-Change (Down)
ALDH1L1	4.22	0.20	LOC283537	7.80	0.19
ALS2CR2	17.84	0.06	LOC441737	2.18	0.28
BST1	5.28	0.11	LOC442258	9.06	0.11
C1orf38	3.34	0.38	LOC647312	4.02	0.23
C9orf119	2.15	0.33	LSM3	3.47	0.19
CERK	2.01	0.46	METTL2A	2.31	0.28
DDI1	2.46	0.27	NULP1	4.70	0.35
DKFZP686M0199	4.86	0.21	OR2A9P	24.83	0.05
DTX2	17.62	0.04	PHTF2	7.63	0.15
ELOF1	2.09	0.47	PRC1	3.37	0.25
FAM86B1	19.09	0.06	PSMD6	18.14	0.09
FASTK	3.20	0.27	RHPN1	7.39	0.12
FGF21	3.72	0.23	ST8SIA1	8.96	0.14
FKBP2	6.38	0.15	TAZ	2.69	0.45
FLJ20280	17.22	0.05	TPD52L3	9.57	0.08
HGD	8.56	0.08	UBAP2	10.77	0.11
KLK15	2.81	0.39	UNQ9438	21.17	0.09
LOC257039	17.01	0.07	WFDC2	12.45	0.08

### FASTK is a Direct Target of miR-106a-5p

**Figure 2:**
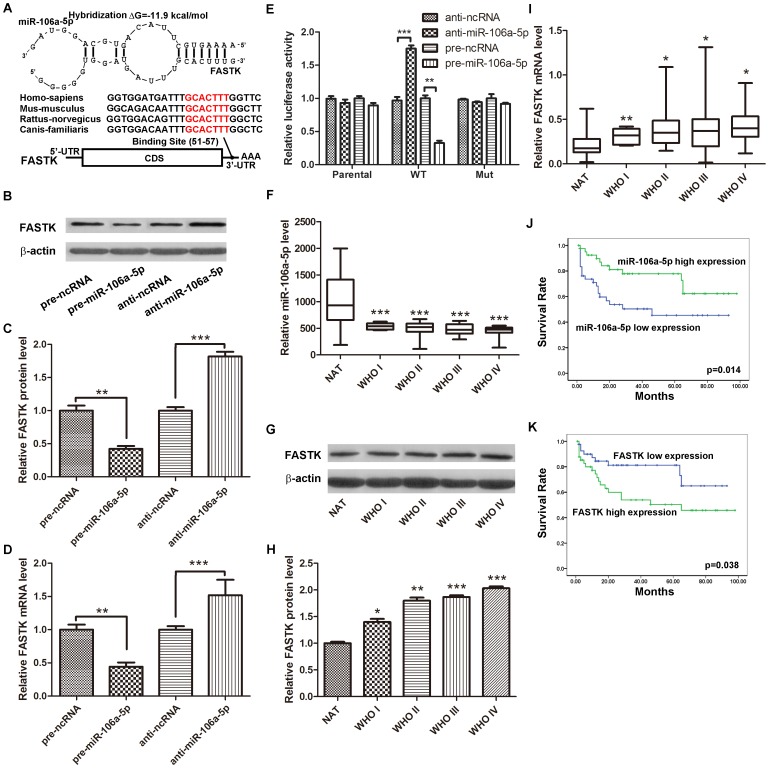
FASTK is a direct target gene of miR-106a-5p. (A) A schematic description of the hypothesized duplexes formed by interactions between the FASTK 3′-UTR binding sites and miR-106a-5p. The predicted free energy of each hybrid is indicated. The complementary seed sites are marked in red, and all of the nucleotides in these regions are completely conserved across several species. (B) Representative western blots showing FASTK protein levels in U251 cells treated with pre-ncRNA, pre-miR-106a-5p, anti-ncRNA and anti-miR-106a-5p. (C) Statistical analysis of three independent experiments. (D) Quantitative real time-PCR analysis of FASTK mRNA expression levels in U251 cells treated with pre-ncRNA, pre-miR-106a-5p, anti-ncRNA and anti-miR-106a-5p. The results shown represent data from three independent experiments. (E) Direct recognition of the FASTK 3′-UTR by miR-106a-5p. Firefly luciferase reporters containing either wt or mut FASTK 3′-UTRs were co-transfected into U251 cells with pre-miR-106a-5p, anti-miR-106a-5p and their corresponding negative controls. The parental luciferase plasmid was also transfected as a control. At 24 h post-transfection, the cells were assayed using luciferase assay kits. The results are presented as the mean ± SD of three independent experiments (** p<0.01; *** p<0.001). (F) Relative miR-106a-5p expression levels in NAT samples and WHO grade I-IV astrocytomas. (G) Representative western blots showing FASTK protein levels in NAT samples and WHO I-IV astrocytomas. (H) Statistical analysis of three independent experiments. (I) Relative FASTK mRNA expression levels in NAT samples and WHO grade I-IV astrocytomas. (J) The relationship between miR-106a-5p expression and astrocytoma patient survival time. (K) The relationship between FASTK expression and astrocytoma patient survival time.

We calculated the probability of whether the differentially regulated genes were predicted miR-106a-5p targets using three widely used programs: TargetScan, PicTar, and microRNA.org. Only the genes predicted as miR-106a-5p targets by at least two of the above-mentioned algorithms were considered positive. Among the list of the 36 genes obtained from the mRNA microarray assay, FASTK (Fas-activated serine/threonine kinase) and PHTF2 (putative homeodomain transcription factor 2) were predicted as miR-106a-5p targets. As FASTK exhibited a higher PicTar score, we sought to verify whether FASTK was a direct target gene of miR-106a-5p. The predicted interaction between miR-106a-5p and its target binding sites within the FASTK 3′-UTR is illustrated in Figure 2A. Since it has been shown that miRNAs expressed at low levels (<100 copies per cell) did not significantly repress target-containing transcripts [28], the absolute expression levels of miR-106a-5p in astrocytoma cells was measured. To calculate the absolute expression levels of miR-106a-5p in astrocytoma cells, a series of synthetic miR-106a-5p oligonucleotides (from 10^−4^ fmol to 10^2^ fmol) were reverse-transcribed and amplified. The absolute amount of miR-106a-5p in astrocytoma cells was then calculated by referring to the standard curve shown in Figure S2 in File S1. miR-106a-5p was present in astrocytoma cells at 1040 copies per cell. At this concentration, it is obviously that miR-106a-5p can efficiently suppress FASTK expression in astrocytoma cells. Firstly, U251 cells were transfected with pre-miR-106a-5p or anti-miR-106a-5p and their corresponding controls. The expression levels of FASTK protein were assessed by western blot analysis at 48 h after transfection. All of the cells transfected with pre-miR-106a-5p exhibited reduced expression of FASTK relative to the cells transfected with pre-ncRNA. In contrast, anti-miR-106a-5p significantly increased the expression of FASTK (Figure 2B). The statistical analysis for FASTK protein expression changes after the overexpression or downregulation of miR-106a-5p in three independent experiments are shown in Figure 2C. Considering that sometimes miRNAs might decrease the levels of a specific target mRNA by affecting its stability, we evaluated the FASTK transcript levels at 24 h after infection. The alterations in FASTK mRNA levels were similar to alterations at the protein level (Figure 2D). These results suggest that miR-106a-5p regulates FASTK expression not only via a post-transcriptional mechanism but also by affecting its mRNA stability. To determine whether the negative regulatory effects of miR-106a-5p on FASTK expression were mediated through binding to the presumed complementary sites at the 3′-UTR of FASTK, we fused the entire FASTK 3′-UTR into a downstream position of a firefly luciferase reporter plasmid. The resulting plasmid was introduced into U251 cells combined with a transfection control plasmid (β-gal), as well as pre-ncRNA, pre-miR-106a-5p, anti-ncRNA or anti-miR-106a-5p. As shown in Figure 2E, miR-106a-5p overexpression significant decreased the luciferase reporter activity (normalized against β-gal activity) compared with the pre-ncRNA treatment, whereas the inhibition of miR-106a-5p significantly increased the reporter activity. However, transfection with the parental luciferase plasmid (without the FASTK 3′-UTR) or with the mutant luciferase plasmid (the FASTK 3′-UTR with mutations in the seed complementary site) did not affect the luciferase reporter activity. This finding suggests that these binding sites significantly contribute to the miRNA:mRNA interaction that mediates the post-transcriptional inhibition of FASTK expression. These results strongly demonstrate that FASTK is the target gene of miR-106a-5p, which directly recognizes the 3′-UTR of the FASTK transcript to downregulate its expression.

Next, the expression of miR-106a-5p and FASTK was evaluated and stratified according to tumor grade in 84 astrocytoma tissues and in 20 NAT samples. As shown in Figure 2F, the expression of miR-106a-5p was progressively decreased from the control samples to the WHO grade IV astrocytomas. As FASTK is the direct target gene of miR-106a-5p, we asked whether FASTK was upregulated in human samples. Figure 2G displays representative western blots of FASTK in astrocytomas. The expression of FASTK in the astrocytomas was significantly higher than that in the NAT samples, and the expression levels increased as the tumor grade increased. The statistical analysis for FASTK protein expression changes from three independent experiments are shown in Figure 2H. As shown in Figure 2I, the mRNA expression levels of FASTK progressively increased from the NAT samples to the grade IV astrocytomas. These results suggested that FASTK is upregulated in astrocytomas and that its upregulation is positively associated with advanced clinical stages. In our previous studies, we found that the reduced expression of miR-106a-5p is significantly associated with poor survival outcome, so we asked whether FASTK upregulation was correlated with patient survival. The expression levels of miR-106a-5p or FASTK were first stratified in the astrocytomas by the median value. Then, the survival outcome of patients with high miR-106a-5p expression levels or high FASTK expression levels (≥ median) was compared with patients exhibiting low miR-106a-5p expression levels or low FASTK expression levels (< median) using Kaplan-Meier survival analysis. Notably, as shown in Figure 2J and Figure 2K, patients with low miR-106a-5p or high FASTK expression levels exhibited poorer survival outcomes than patients with high miR-106a-5p or low FASTK expression levels (p = 0.014 and p = 0.038, respectively). These results further confirm the negative regulation of FASTK by miR-106a-5p *in vivo*.

### The Role of miR-106a-5p Targeting of FASTK in Cell Proliferation, Migration and Apoptosis

**Figure 3:**
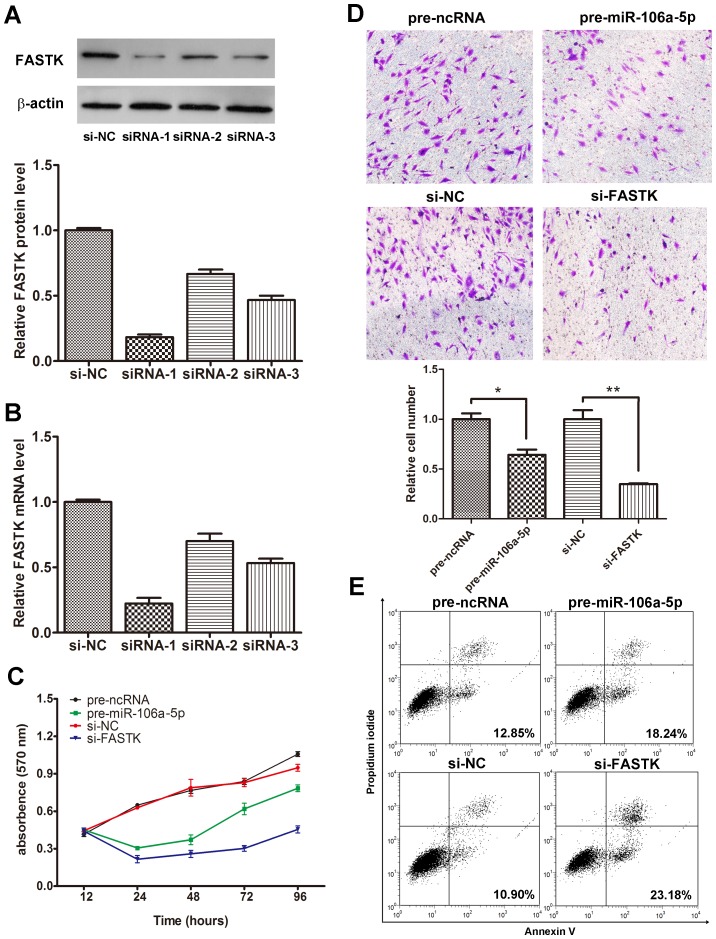
The role of miR-106a-5p and FASTK in cell proliferation, migration and apoptosis. FASTK siRNA interference assay (A–B). Three siRNA sequences targeting different sites of human FASTK cDNA and a scrambled control siRNA (si-NC) were transfected into U251 cells using Lipofectamine 2000. Total protein or total RNA was isolated at 48 h or 24 h post-transfection. FASTK protein levels were determined by western blot analysis (A), and FASTK mRNA levels were assessed by qRT-PCR (B). The siRNA eliciting the most optimal interfering effect (siRNA-1, named si-FASTK) was used in further studies. (C) The role of miR-106a-5p and FASTK on cell proliferation. An MTT cell viability assay was performed at 12, 24, 48, 72 and 96 h after transfection of U251 cells with equal concentrations of pre-ncRNA, pre-miR-106a-5p, si-NC and si-FASTK. (D) Transwell assays of U251 cells treated with equal concentrations of pre-ncRNA, pre-miR-106a-5p, si-NC and si-FASTK. The images shown are representative images from three independent experiments, and a statistical analysis was performed (mean ± SD; * p<0.05, ** p<0.01). (E) The role of miR-106a-5p and FASTK on apoptosis. U251 cells were transfected with equal concentrations of pre-ncRNA, pre-miR-106a-5p, si-NC and si-FASTK. The experiment was repeated three times, and representative data are shown.

To investigate the cellular phenotypes triggered by the targeting of FASTK by miR-106a-5p, U251 cells were transfected with pre-miR-106a-5p and siRNA against FASTK (si-FASTK) and analyzed for the changes in proliferation, migration, and apoptosis. Cells transfected with pre-ncRNA or control siRNA (si-NC) served as controls. The efficient depletion of the FASTK protein and mRNA expression levels is shown in Figure 3A and Figure 3B. As siRNA-1 elicited the most efficient inhibition, it was used in the subsequent experiments. The MTT assay and growth curves revealed that the cells that were transiently transfected with pre-miR-106a-5p proliferated at a significantly reduced rate compared to the pre-ncRNA-transfected cells (Figure 3C). The relative cell survival rate of the pre-miR-106a-5p-transfected cells at 96 h was 74.4%. Our proliferation assay showed that the knockdown of FASTK gene expression significantly inhibited cell proliferation (Figure 3C). Notably, the inhibitory effect induced by si-FASTK was stronger than that induced by pre-miR-106a-5p.

Cell migration is an important aspect of cancer progression, involving the invasion of tumor cells into contiguous tissues and the dissolution of extracellular matrix proteins. To investigate whether miR-106a-5p elicits a direct functional role in facilitating astrocytoma cell migration, we evaluated cancer cell migration using a transwell-based assay. As shown in Figure 3D, the overexpression of miR-106a-5p induced by transfection of pre-miR-106a-5p reduced the migration of U251 cells by approximately 30% compared to control-transfected cells, whereas the knockdown of FASTK significantly suppressed the ability of astrocytoma cells to migrate through non-matrigel-coated membranes by approximately 65%. The inhibition of migration induced by the interference of FASTK was also stronger than that elicited by miR-106a-5p overexpression.

Next, we used Annexin V and PI double-staining FACS analysis to investigate the effects of miR-106a-5p and FASTK on the apoptosis of astrocytoma cells. As shown in Figure 3E, the overexpression of miR-106a-5p induced by transfection with pre-miR-106a-5p resulted in a significant increase in apoptotic cells compared with the negative control-transfected cells. Treatment with si-FASTK for 48 h also increased apoptosis and the numbers of necrotic cells. Furthermore, the apoptotic rate was much higher when si-FASTK was transfected compared to when pre-miR-106a-5p was transfected. Furthermore, the results were similar in U87 cells, as shown in Figure S3 in File S1.

## Discussion

In recent years, the deregulation of miRNAs has been identified to play an active role in cancer development [29]. miRNAs serve functionally as “oncogenes” or “tumor suppressor genes” in tumorigenesis and regulate multiple cellular processes involved in cancer progression [30]. Although the number of cancer-related miRNAs continues to increase, information regarding their precise cellular function remains limited. One of the main challenges in understanding the functions of miRNAs is the identification of their actual target genes.

In our previous study, we established a unique molecular diagnostic signature for astrocytomas including miR-21, miR-24, miR-30c, miR-106a-5p, miR-124, miR-137, and miR-181b [8]. miR-106a-5p is one of the most significantly downregulated miRNAs in astrocytomas, and its low expression is significantly associated with poor survival outcome, which triggered our interest in investigating its function and its target genes during astrocytoma development. miR-106a-5p, which is located on Xq26.2, belongs to the miR-17 family, whose overexpression has been detected in various types of human cancer. The members of this family have been found to facilitate cancer development by promoting cell proliferation, inhibiting apoptosis and inducing tumor angiogenesis [31], [32]. However, the potential role of miR-106a-5p as an oncogene or a tumor suppressor in cancer development remains controversial. miR-106a-5p increases cell migration and invasion by inhibiting the anti-metastatic gene TGFBR2 (transforming growth factor-β receptor 2) in colorectal cancer [16], inhibits the cellular extrinsic apoptotic pathway by targeting FAS in gastric cancer [10], and promotes cell growth and invasion by targeting ZBTB4 (zinc-finger and BTB domain containing 4) in breast cancer [14]. In contrast, miR-106a-5p inhibits cell proliferation and induces apoptosis by targeting E2F1 (E2F transcription factor 1) in gliomas [20]. However, there are few reports about miR-106a-5p function and its target genes as a tumor suppressor, particularly in astrocytomas. In this study, we found that the overexpression of miR-106a-5p significantly inhibited astrocytoma cell proliferation and migration and promoted apoptosis, which supports the role of miR-106a-5p as a tumor suppressor in astrocytomas. As every coin has two sides, the divergent function of miR-106a-5p as an oncogene or a tumor suppressor in cancer development might depend on the tumor type, and further investigation is required to better understand this issue.

In this study, FASTK was experimentally validated as a miR-106a-5p target. FASTK belongs to the serine/threonine protein kinase family and is a potential regulator of Fas-induced apoptosis. FASTK was previously identified as a TIA1-interacting protein in a yeast two-hybrid screen [33]. TIA1 is an RNA-binding protein that serves as a downstream effector of the PKR/eIF2α translational control pathway [34]. In response to Fas ligation, FASTK is rapidly dephosphorylated, and TIA1 is concomitantly phosphorylated on serine residues [33]. FASTK reportedly tethers to mitochondria via a lysine/arginine-rich domain at its carboxyl terminus, through which FASTK interacts with BCL-X_L_. The FASTK-BCL-X_L_ interactions are likely to regulate mitochondrial metabolism during Fas-induced apoptosis [35]. In cells exposed to environmental stress, FASTK moves to stress granules, where it interacts with TIA1 to modulate the process of stress-induced translational silencing [36]. Furthermore, FASTK is an antiapoptotic protein that senses mitochondrial stress, modulates a TIA1-regulated posttranscriptional stress response program, and synergizes with TIA1/TIAR proteins to regulate Fas alternative splicing [37], [38]. Fas-mediated apoptosis is characterized by FASTK dephosphorylation and TIA1 phosphorylation. Both proteins are activated, and FASTK-mediated TIA1 activation plays a key role in apoptosis. The reduced proliferation, decreased migration and enhanced apoptosis induced by the depletion of FASTK support the role of FASTK as an antiapoptotic factor. Notably, the knockdown of FASTK can significantly increase the total neurite length in SH-SY-5Y cells [39]. In our previous work, we found that activated β-catenin can force N2A cell-derived neurons back to tumor-like neuroblasts and plays an important role in neuroblastoma formation [26]. Thus, FASTK might functionally contribute to astrocytoma progression in roles other than simply as an anti-apoptotic protein.

In our study, miR-106a-5p directly targets FASTK expression in astrocytoma cells and they play important roles in the disease progression. Overexpression of miR-106a-5p and corresponding decreased FASTK expression, decreased oncogenic potential of cells, as evidenced by decreased proliferation rate, cell migration and increased apoptosis damage. The mechanism by which miR-106a-5p decreases oncogenic potential of cells is most likely through inhibition of FASTK, a potential regulator of Fas-induced apoptosis. Fas (CD95/APO-1) is a member of the tumor necrosis factor/nerve growth factor (TNF/NGF) superfamily [40]. The interaction of Fas with Fas ligand (FasL) allows the formation of a death-inducing signaling complex that includes Fas-associated death domain protein (FADD), caspase-8 and caspase-10 [41], [42]. The autoproteolytic processing of the caspases in the complex triggers a downstream caspase cascade and leads to apoptosis. Signaling through Fas has been shown to activate the three main mitogen-activated protein kinase (MAPK) pathways, p38, JNK1/2, and ERK1/2, as well as the transcription factor NF-κB, leading to cell proliferation, migration and inflammation [43]. The reduced proliferation, decreased migration and enhanced apoptosis induced by the depletion of FASTK support the role of FASTK as an antiapoptotic factor. We may suggest that the tumor suppressive potential of miR-106a-5p is due to inhibition of FASTK which interacts with TIA1 to mediate Fas-induced apoptosis during astrocytoma pathogenesis. To the best of our knowledge, this study is the first report showing direct regulation of FASTK by miR-106a-5p.

In summary, our data indicate that miR-106a-5p is a tumor suppressor gene in astrocytomas. The overexpression of miR-106a-5p inhibits astrocytoma cell proliferation, migration, and invasion and promotes apoptosis. Moreover, we show that FASTK is a direct target for miR-106a-5p. Although much remains to be elucidated in terms of the role of miR-106a-5p in the pathogenesis of astrocytomas, miR-106a-5p represents a new potential therapeutic target for the treatment of astrocytomas.

## Supporting Information

File S1 **Supporting information file including Figures S1–S3 and Table S1. Figure S1.** Relative expression of miR-106b-5p after miR-106a-5p transfection. U251 cells were seeded into 6-well plates and transfected the following day using Lipofectamine 2000. For each well, 100 pmol of pre-ncRNA, pre-miR-106a-5p, anti-ncRNA or anti-miR-106a-5p was transfected. The intercellular levels of miR-106b-5p were evaluated by qRT-PCR at 24 h after transfection. For comparison, the expression levels of miR-106b-5p in pre-ncRNA- or anti-ncRNA-transfected cells were arbitrarily set at 1. The results are presented as the mean ± SD of three independent experiments. **Figure S2.** Evaluation of the absolute expression level of miR-106a-5p in astrocytoma cells. Either 10^−4^, 10^−3^, 10^−2^, 10^−1^, 10^0^, 10^1^, or 10^2^ fmol of single strand miR-106a-5p synthesized by TaKaRa (Dalian, China) were assessed by qRT-PCR assay. The resulting Ct values were plotted versus the log_10_ of the amount of input miR-106a-5p. Then the absolute amount of miR-106a-5p in astrocytoma cells was calculated by referring to the standard curve. **Figure S3.** The role of miR-106a-5p and FASTK in cell apoptosis in U87 cells. U87 cells were transfected with equal concentrations of pre-ncRNA, pre-miR-106a-5p, si-NC and si-FASTK. The experiment was repeated three times, and representative data are shown. **Table S1.** Summary of the demographic and clinical features of the 84 astrocytoma samples and the 20 NAT samples
(DOC)

## References

[1] Wen PY, Kesari S (2008) Malignant gliomas in adults. N Engl J Med 359: 492–507. 18669428 10.1056/NEJMra0708126

[2] Gabayan AJ, Green SB, Sanan A, Jenrette J, Schultz C, et al.. (2006) GliaSite brachytherapy for treatment of recurrent malignant gliomas: a retrospective multi-institutional analysis. Neurosurgery 58: 701–709; discussion 701–709. 10.1227/01.NEU.0000194836.07848.69 16575334

[3] Hatanpaa KJ, Burma S, Zhao D, Habib AA (2010) Epidermal growth factor receptor in glioma: signal transduction, neuropathology, imaging, and radioresistance. Neoplasia 12: 675–684. 20824044 10.1593/neo.10688 PMC2933688

[4] Endersby R, Baker SJ (2008) PTEN signaling in brain: neuropathology and tumorigenesis. Oncogene 27: 5416–5430. 18794877 10.1038/onc.2008.239

[5] Bartel DP (2004) MicroRNAs: genomics, biogenesis, mechanism, and function. Cell 116: 281–297. 14744438 10.1016/s0092-8674(04)00045-5

[6] Ryan BM, Robles AI, Harris CC (2010) Genetic variation in microRNA networks: the implications for cancer research. Nat Rev Cancer 10: 389–402. 20495573 10.1038/nrc2867 PMC2950312

[7] Lu J, Getz G, Miska EA, Alvarez-Saavedra E, Lamb J, et al (2005) MicroRNA expression profiles classify human cancers. Nature 435: 834–838. 15944708 10.1038/nature03702

[8] Zhi F, Chen X, Wang S, Xia X, Shi Y, et al (2010) The use of hsa-miR-21, hsa-miR-181b and hsa-miR-106a as prognostic indicators of astrocytoma. Eur J Cancer 46: 1640–1649. 20219352 10.1016/j.ejca.2010.02.003

[9] Kim BH, Hong SW, Kim A, Choi SH, Yoon SO (2012) Prognostic implications for high expression of oncogenic microRNAs in advanced gastric carcinoma. J Surg Oncol. 10.1002/jso.23271 22996433

[10] Wang Z, Liu M, Zhu H, Zhang W, He S, et al.. (2012) miR-106a Is frequently upregulated in gastric cancer and inhibits the extrinsic apoptotic pathway by targeting FAS. Mol Carcinog. 10.1002/mc.21899 22431000

[11] Yao Y, Suo AL, Li ZF, Liu LY, Tian T, et al (2009) MicroRNA profiling of human gastric cancer. Mol Med Report 2: 963–970. 10.3892/mmr_00000199 21475928

[12] Xiao B, Guo J, Miao Y, Jiang Z, Huan R, et al (2009) Detection of miR-106a in gastric carcinoma and its clinical significance. Clin Chim Acta 400: 97–102. 18996365 10.1016/j.cca.2008.10.021

[13] Guo J, Miao Y, Xiao B, Huan R, Jiang Z, et al (2009) Differential expression of microRNA species in human gastric cancer versus non-tumorous tissues. J Gastroenterol Hepatol 24: 652–657. 19175831 10.1111/j.1440-1746.2008.05666.x

[14] Kim K, Chadalapaka G, Lee SO, Yamada D, Sastre-Garau X, et al (2012) Identification of oncogenic microRNA-17–92/ZBTB4/specificity protein axis in breast cancer. Oncogene 31: 1034–1044. 21765466 10.1038/onc.2011.296 PMC3288192

[15] Wang F, Zheng Z, Guo J, Ding X (2010) Correlation and quantitation of microRNA aberrant expression in tissues and sera from patients with breast tumor. Gynecol Oncol 119: 586–593. 20801493 10.1016/j.ygyno.2010.07.021

[16] Feng B, Dong TT, Wang LL, Zhou HM, Zhao HC, et al (2012) Colorectal Cancer Migration and Invasion Initiated by microRNA-106a. PLoS One 7: e43452. 22912877 10.1371/journal.pone.0043452 PMC3422256

[17] Donnem T, Fenton CG, Lonvik K, Berg T, Eklo K, et al (2012) MicroRNA signatures in tumor tissue related to angiogenesis in non-small cell lung cancer. PLoS One 7: e29671. 22295063 10.1371/journal.pone.0029671 PMC3266266

[18] Hummel R, Hussey DJ, Michael MZ, Haier J, Bruewer M, et al (2011) MiRNAs and their association with locoregional staging and survival following surgery for esophageal carcinoma. Ann Surg Oncol 18: 253–260. 20628822 10.1245/s10434-010-1213-y

[19] Diaz R, Silva J, Garcia JM, Lorenzo Y, Garcia V, et al (2008) Deregulated expression of miR-106a predicts survival in human colon cancer patients. Genes Chromosomes Cancer 47: 794–802. 18521848 10.1002/gcc.20580

[20] Yang G, Zhang R, Chen X, Mu Y, Ai J, et al.. (2011) MiR-106a inhibits glioma cell growth by targeting E2F1 independent of p53 status. J Mol Med. 10.1007/s00109-011-0775-x 21656380

[21] Chen C, Ridzon DA, Broomer AJ, Zhou Z, Lee DH, et al (2005) Real-time quantification of microRNAs by stem-loop RT-PCR. Nucleic Acids Res 33: e179. 16314309 10.1093/nar/gni178 PMC1292995

[22] Tusher VG, Tibshirani R, Chu G (2001) Significance analysis of microarrays applied to the ionizing radiation response. Proc Natl Acad Sci U S A 98: 5116–5121. 11309499 10.1073/pnas.091062498 PMC33173

[23] Lewis BP, Shih IH, Jones-Rhoades MW, Bartel DP, Burge CB (2003) Prediction of mammalian microRNA targets. Cell 115: 787–798. 14697198 10.1016/s0092-8674(03)01018-3

[24] Krek A, Grun D, Poy MN, Wolf R, Rosenberg L, et al (2005) Combinatorial microRNA target predictions. Nat Genet 37: 495–500. 15806104 10.1038/ng1536

[25] John B, Enright AJ, Aravin A, Tuschl T, Sander C, et al (2004) Human MicroRNA targets. PLoS Biol 2: e363. 15502875 10.1371/journal.pbio.0020363 PMC521178

[26] Zhi F, Gong G, Xu Y, Zhu Y, Hu D, et al (2012) Activated beta-catenin Forces N2A Cell-derived Neurons Back to Tumor-like Neuroblasts and Positively Correlates with a Risk for Human Neuroblastoma. Int J Biol Sci 8: 289–297. 22298956 10.7150/ijbs.3520 PMC3269611

[27] Wang K, Li P, Dong Y, Cai X, Hou D, et al (2011) A microarray-based approach identifies ADP ribosylation factor-like protein 2 as a target of microRNA-16. J Biol Chem 286: 9468–9476. 21199864 10.1074/jbc.M110.178335 PMC3058993

[28] Brown BD, Gentner B, Cantore A, Colleoni S, Amendola M, et al (2007) Endogenous microRNA can be broadly exploited to regulate transgene expression according to tissue, lineage and differentiation state. Nat Biotechnol 25: 1457–1467. 18026085 10.1038/nbt1372

[29] Calin GA, Croce CM (2006) MicroRNA signatures in human cancers. Nat Rev Cancer 6: 857–866. 17060945 10.1038/nrc1997

[30] Chitwood DH, Timmermans MC (2010) Small RNAs are on the move. Nature 467: 415–419. 20864994 10.1038/nature09351

[31] Mendell JT (2008) miRiad roles for the miR-17–92 cluster in development and disease. Cell 133: 217–222. 18423194 10.1016/j.cell.2008.04.001 PMC2732113

[32] Olive V, Jiang I, He L (2010) mir-17–92, a cluster of miRNAs in the midst of the cancer network. Int J Biochem Cell Biol 42: 1348–1354. 20227518 10.1016/j.biocel.2010.03.004 PMC3681296

[33] Tian Q, Taupin J, Elledge S, Robertson M, Anderson P (1995) Fas-activated serine/threonine kinase (FAST) phosphorylates TIA-1 during Fas-mediated apoptosis. J Exp Med 182: 865–874. 7544399 10.1084/jem.182.3.865 PMC2192163

[34] Kedersha NL, Gupta M, Li W, Miller I, Anderson P (1999) RNA-binding proteins TIA-1 and TIAR link the phosphorylation of eIF-2 alpha to the assembly of mammalian stress granules. J Cell Biol 147: 1431–1442. 10613902 10.1083/jcb.147.7.1431 PMC2174242

[35] Li W, Kedersha N, Chen S, Gilks N, Lee G, et al (2004) FAST is a BCL-X(L)-associated mitochondrial protein. Biochem Biophys Res Commun 318: 95–102. 15110758 10.1016/j.bbrc.2004.03.188

[36] Simarro M, Mauger D, Rhee K, Pujana MA, Kedersha NL, et al (2007) Fas-activated serine/threonine phosphoprotein (FAST) is a regulator of alternative splicing. Proc Natl Acad Sci U S A 104: 11370–11375. 17592127 10.1073/pnas.0704964104 PMC2040905

[37] Li W, Simarro M, Kedersha N, Anderson P (2004) FAST is a survival protein that senses mitochondrial stress and modulates TIA-1-regulated changes in protein expression. Mol Cell Biol 24: 10718–10732. 15572676 10.1128/MCB.24.24.10718-10732.2004 PMC533970

[38] Izquierdo JM, Valcarcel J (2007) Fas-activated serine/threonine kinase (FAST K) synergizes with TIA-1/TIAR proteins to regulate Fas alternative splicing. J Biol Chem 282: 1539–1543. 17135269 10.1074/jbc.C600198200

[39] Loh SH, Francescut L, Lingor P, Bahr M, Nicotera P (2008) Identification of new kinase clusters required for neurite outgrowth and retraction by a loss-of-function RNA interference screen. Cell Death Differ 15: 283–298. 18007665 10.1038/sj.cdd.4402258

[40] Trauth BC, Klas C, Peters AM, Matzku S, Moller P, et al (1989) Monoclonal antibody-mediated tumor regression by induction of apoptosis. Science 245: 301–305. 2787530 10.1126/science.2787530

[41] Salvesen GS, Dixit VM (1999) Caspase activation: the induced-proximity model. Proc Natl Acad Sci U S A 96: 10964–10967. 10500109 10.1073/pnas.96.20.10964 PMC34227

[42] Lee KH, Feig C, Tchikov V, Schickel R, Hallas C, et al (2006) The role of receptor internalization in CD95 signaling. EMBO J 25: 1009–1023. 16498403 10.1038/sj.emboj.7601016 PMC1409734

[43] Brint E, O’Callaghan G, Houston A (2013) Life in the Fas lane: differential outcomes of Fas signaling. Cell Mol Life Sci. 10.1007/s00018-013-1327-z PMC11113183 23579628

