# Supplementary material for: miR-106a-5p Inhibits the Proliferation and Migration of Astrocytoma Cells and Promotes Apoptosis by Targeting FASTK
Source: PLoS One. 2013 Aug 27;8(8):e72390. doi: 10.1371/journal.pone.0072390 (PMC3754986; doi:10.1371/journal.pone.0072390)
Supplement: File S1 — Supporting information file including Figures S1–S3 and Table S1. Figure S1. Relative expression of miR-106b-5p after miR-106a-5p transfection. U251 cells were seeded into 6-well plates and transfected the following day using Lipofectamine 2000. For each well, 100 pmol of pre-ncRNA, pre-miR-106a-5p, anti-ncRNA or anti-miR-106a-5p was transfected. The intercellular levels of miR-106b-5p were evaluated by qRT-PCR at 24 h after transfection. For comparison, the expression levels of miR-106b-5p in pre-ncRNA- or anti-ncRNA-transfected cells were arbitrarily set at 1. The results are presented as the mean ± SD of three independent experiments. Figure S2. Evaluation of the absolute expression level of miR-106a-5p in astrocytoma cells. Either 10−4, 10−3, 10−2, 10−1, 100, 101, or 102 fmol of single strand miR-106a-5p synthesized by TaKaRa (Dalian, China) were assessed by qRT-PCR assay. The resulting Ct values were plotted versus the log10 of the amount of input miR-106a-5p. Then the absolute amount of miR-106a-5p in astrocytoma cells was calculated by referring to the standard curve. Figure S3. The role of miR-106a-5p and FASTK in cell apoptosis in U87 cells. U87 cells were transfected with equal concentrations of pre-ncRNA, pre-miR-106a-5p, si-NC and si-FASTK. The experiment was repeated three times, and representative data are shown. Table S1. Summary of the demographic and clinical features of the 84 astrocytoma samples and the 20 NAT samples (DOC) [file pone.0072390.s001.doc]

**Supporting Information**

**miR-106a-5p inhibits the proliferation and migration of astrocytoma cells and promotes apoptosis by targeting FASTK**

Feng Zhi^1,#^, Guangxin Zhou^3,#^, Naiyuan Shao^2,#^, Xiwei Xia^2^, Yimin Shi^2^, Qiang Wang^2^, Yi Zhang^2^, Rong Wang^1^, Lian Xue^1^, Suinuan Wang^2^, Sujia Wu^3,*^, Ya Peng^2*^, Yilin Yang^1*^

^1^ Modern Medical Research Center, Third Affiliated Hospital of Soochow University, Changzhou, Jiangsu, China;

^2^ Department of Neurosurgery, Third Affiliated Hospital of Soochow University, Changzhou, Jiangsu, China;

^3^ Department of Orthopedics, Jinling Hospital, School of Medicine, Nanjing University, Nanjing, Jiangsu, China.

**Key words:** miR-106a-5p, FASTK, astrocytoma, prognosis, proliferation, migration, apoptosis

# These authors contributed equally in this work.

*Corresponding authors: Yilin Yang^a^, Ya Peng^b^ and Suinuan Wang^c^

^a^E-mail: yilinyang.czfph@gmail.com

^b^E-mail: yapeng997@163.com

^c^E-mail: sujiawu1957@yahoo.cn

**Figure S1.** Relative expression of miR-106b-5p after miR-106a-5p transfection. U251 cells were seeded into 6-well plates and transfected the following day using Lipofectamine 2000. For each well, 100 pmol of pre-ncRNA, pre-miR-106a-5p, anti-ncRNA or anti-miR-106a-5p was transfected. The intercellular levels of miR-106b-5p were evaluated by qRT-PCR at 24 h after transfection. For comparison, the expression levels of miR-106b-5p in pre-ncRNA- or anti-ncRNA-transfected cells were arbitrarily set at 1. The results are presented as the mean ± SD of three independent experiments.


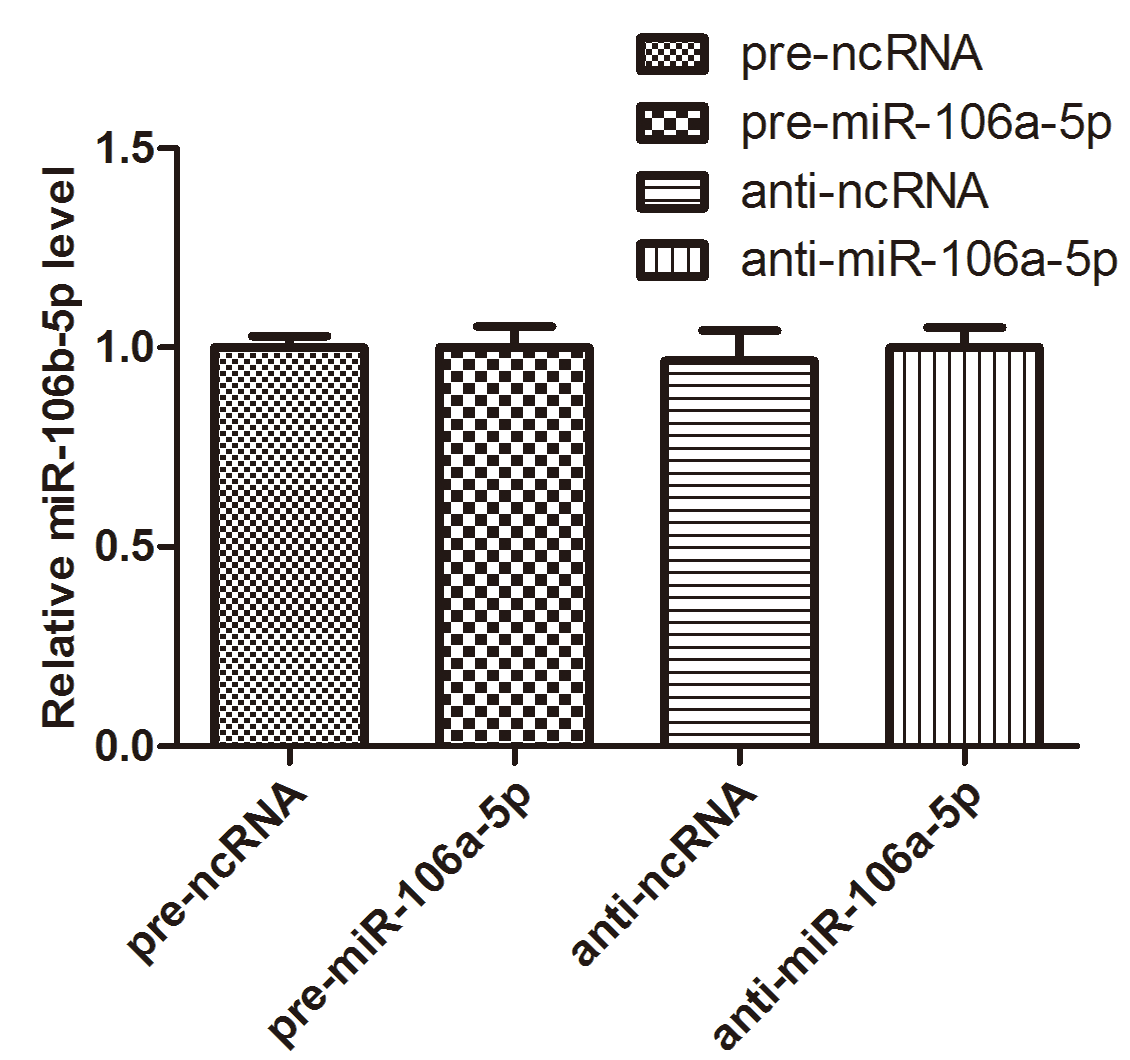


**Figure S2.** Evaluation of the absolute expression level of miR-106a-5p in astrocytoma cells. Either 10^-4^, 10^-3^, 10^-2^, 10^-1^, 10^0^, 10^1^, or 10^2^ fmol of single strand miR-106a-5p synthesized by TaKaRa (Dalian, China) were assessed by qRT-PCR assay. The resulting Ct values were plotted versus the log_10_ of the amount of input miR-106a-5p. Then the absolute amount of miR-106a-5p in astrocytoma cells was calculated by referring to the standard curve.


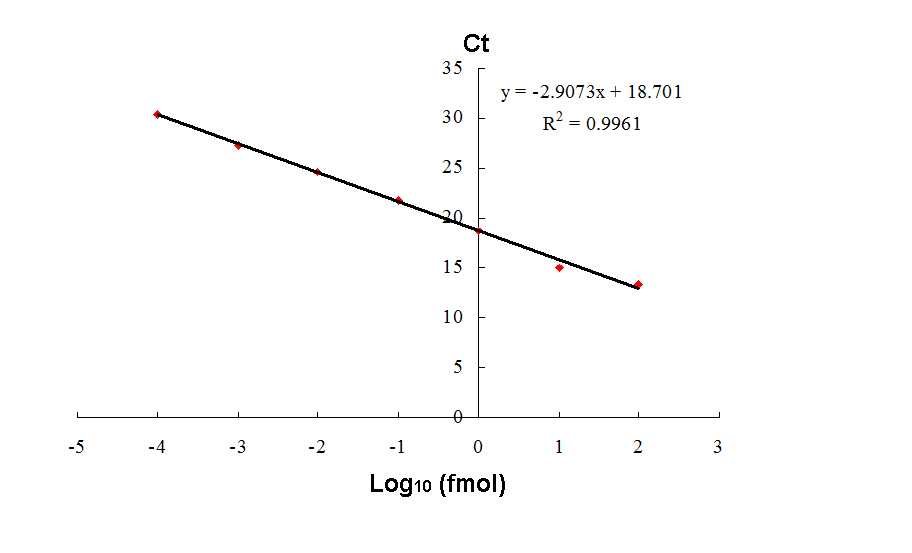


**Figure S3.** The role of miR-106a-5p and FASTK in cell apoptosis in U87 cells. U87 cells were transfected with equal concentrations of pre-ncRNA, pre-miR-106a-5p, si-NC and si-FASTK. The experiment was repeated three times, and representative data are shown.


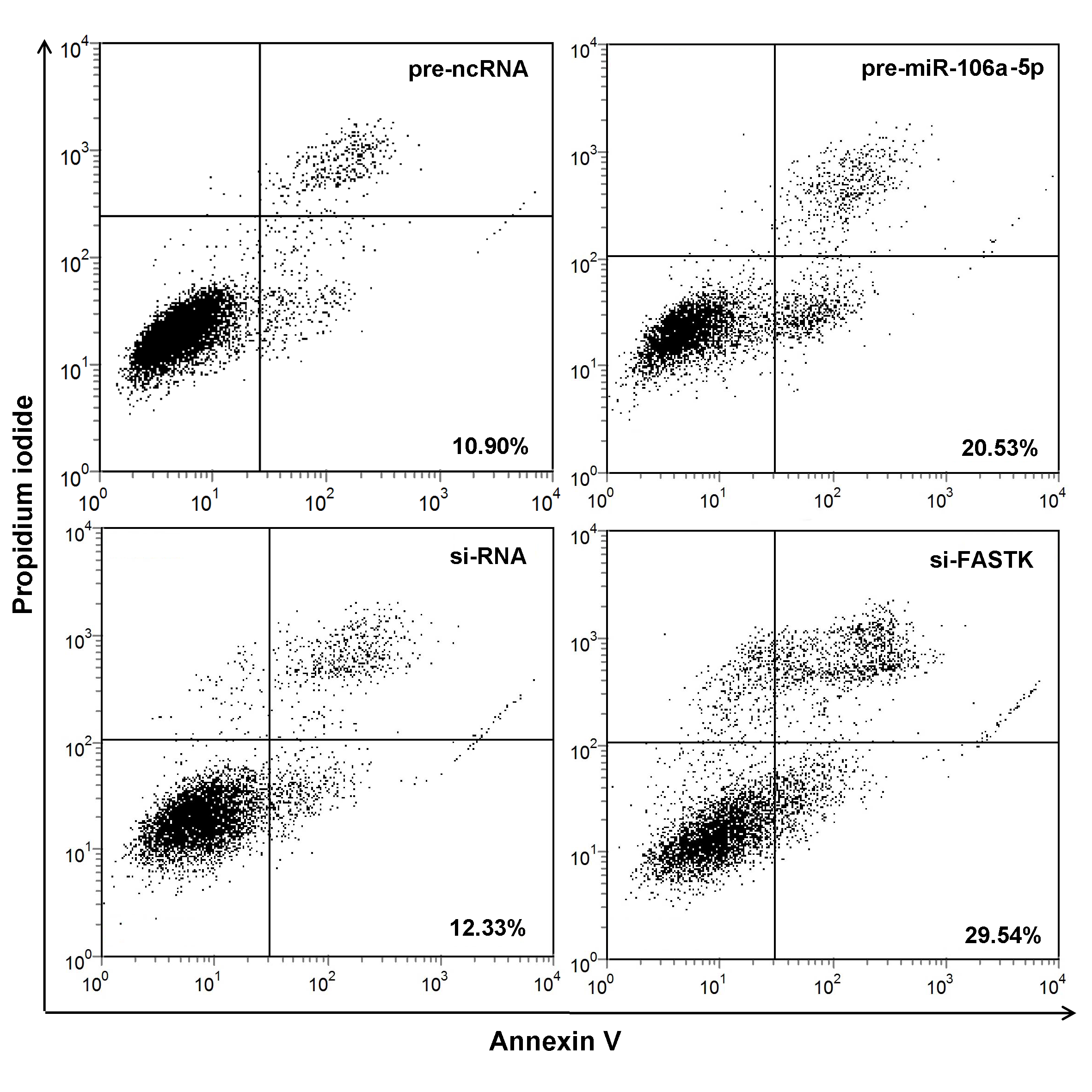


**Table S1.** Summary of the demographic and clinical features of the 84 astrocytoma samples and the 20 NAT samples

| **Variable** | Astrocytoma (n=84) | | Control (n=20) | |
| --- | --- | --- | --- | --- |
|  | No. | % | No. | % |
| **Age (years)** |  |  |  |  |
| ≤ 49 | 48 | 57.1 | 12 | 60 |
| > 49 | 36 | 42.9 | 8 | 40 |
| **Sex** |  |  |  |  |
| Male | 46 | 54.8 | 12 | 60 |
| Female | 38 | 45.2 | 8 | 40 |
| **WHO grade** |  |  |  |  |
| Pilocytic astrocytoma (WHO grade I) | 6 | 7.1 |  |  |
| Diffuse astrocytoma (WHO grade II) | 26 | 31 |  |  |
| Anaplastic astrocytoma (WHO grade III) | 31 | 36.9 |  |  |
| Glioblastoma multiforme (WHO grade IV) | 21 | 25 |  |  |
| **Follow-up** |  |  |  |  |
| Alive | 53 | 63.1 |  |  |
| Dead | 31 | 36.9 |  |  |
| Mean survival time (months) | 64.9 ± 4.8 | |  |  |
